# Non-Isothermal Crystallisation Kinetics of Polypropylene at High Cooling Rates and Comparison to the Continuous Two-Domain pvT Model

**DOI:** 10.3390/polym12071515

**Published:** 2020-07-08

**Authors:** Jonathan Alms, Christian Hopmann, Jian Wang, Tobias Hohlweck

**Affiliations:** 1 Institute for Plastics Processing (IKV), RWTH Aachen University, Aachen 52074, Germany; office@ikv.rwth-aachen.de (C.H.); tobias.hohlweck@ikv.rwth-aachen.de (T.H.); 2 College of Mechanical and Electrical Engineering, Beijing University of Chemical Technology, Beijing 100029, China

**Keywords:** polypropylene, material model, crystallisation kinetics, pvT behaviour, differential scanning calorimetry

## Abstract

The modelling of the correlation between pressure, specific volume and temperature (pvT) of polymers is highly important for applications in the polymer processing of semi-crystalline thermoplastics, especially in injection moulding. In injection moulding, the polymer experiences a wide range of cooling rates, for example, 60 °C/min near the centre of the part and up to 3000 °C/min near the mould walls. The cooling rate has a high influence on the pvT behaviour, as was shown in the continuous two-domain pvT model (CTD). This work examined the Hoffman–Lauritzen nucleation and growth theory used in the modified Hammami model for extremely high cooling rates (up to 300,000 °C/min) by means of Flash differential scanning calorimeter (DSC) measurements. The results were compared to those of the empirical continuous two-domain pvT model. It is shown that the Hammami model is not suitable to predict the crystallisation kinetics of polypropylene at cooling rates above 600 °C/min, but that the continuous two-domain pvT model is well able to predict crystallisation temperatures at high cooling rates.

## 1. Introduction

The prediction of the correlation between pressure, specific volume and temperature (pvT) of polymers is highly important for applications in the polymer processing of semi-crystalline thermoplastics, especially in injection moulding. Current models that predict shrinkage and warpage, which are important quality features of injection moulded parts, are all based on pvT models [1,2,3,4].

The Tait model is widely used for polymers because of its high fit accuracy and simple form [5,6,7,8,9]. It is used in commercial simulation software, such as Moldflow (Autodesk, Inc., Mill Valley, CA, USA), Moldex 3D (CoreTech System Co., Ltd., Zhubei City, Taiwan), Sigmasoft (SIGMA Engineering GmbH, Aachen, Germany) and Cadmould (Simcon kunststofftechnische Software GmbH, Würselen, Germany). Wang et al. [10] improved the Tait model to even higher fitting accuracy due to the consideration of second-order polynomials. It also removes the discontinuity at the transition temperature between the models for the molten and solid states (two domains). This leads to accurate data prediction during the injection moulding simulation, since a small change in temperature around the transition temperature causes significant changes in the specific volume. The discontinuity is solved by enforcing the model of both states to be equal at the transition temperature. Therefore, the continuous two-domain pvT model (CTD) was developed, which also considers the effects of the cooling rate on the pvT behaviour [10].

The CTD is an empirical model, which was fitted to pvT data measured on an isotactic polypropylene applying pressures from 200 to 2200 bar and cooling rates up to 20 °C/min. This is problematic for modelling injection moulding processes since large cooling rates up to 3000 °C/min close to the mould walls and 60 °C/min near the centre of the part are usually experienced [11]. Since the cooling rate has a clear influence on the pvT behaviour of polymers and their solidification process, it is important to have an accurate description of the pvT behaviour at process-relevant cooling rates [9,12,13,14,15,16]. The extrapolation of the CTD to cooling rates more than 100 times larger is difficult. Therefore, a combined model using the physical description of polymer solidification at pressures of 1 bar and cooling rates between 2 °C/min and 300,000 °C/min and the pressure dependency found in the CTD should provide a description of the pvT behaviour at high pressures (from CTD) and high cooling rates (from a physical model). Therefore, a crystallisation kinetics model was adopted to provide the physical description at low pressure and its results were compared with the extrapolations of the CTD.

Crystallisation kinetics models are usually based on the Avrami model, which describes time evolution of relative crystallinity in polymer melts during cooling [17]. The Avrami model is an isothermal description of the cooling process [18]. Since cooling processes tend to be non-isothermal in nature, the Avrami model has been improved and expanded on many occasions to incorporate a non-isothermal physical description of the crystallisation process [19,20,21,22,23,24,25]. It was found that the model proposed by Hammami et al. [24] shows the best description of the solidification over a wide range of cooling rates [18]. Mubarak et al. [18] modified the Hammami model by introducing the non-isothermal induction time. They studied the modified model at cooling rates up to 100 °C/min, which is at least 30 times smaller than needed for the description of injection moulding processes.

This work expanded on the works of Mubarak et al. by applying the Hammami model to a range of cooling rates of 2 to 6000 °C/min and compared the results of the physical Hammami model to those of the empirical CTD at the transition temperature. The CTD was then further expanded by using the data measured in this work at the pressure of 1 bar in the fit data. 

In order to apply the Hammami model, the Avrami exponent was determined first to characterise the crystallisation type, followed by a determination of the influence of the thermal history on the transition temperature by means of differential scanning calorimeter (DSC) and Flash DSC measurements. To use Mubarak et al.’s approach on the Hammami model, the non-isothermal induction time was calculated and finally the Hammami model was used to predict the non-isothermal transition temperatures at different cooling rates. The prediction was then compared to the CTD.

## 2. Experiments

### 2.1. Materials

In all experiments, an isotactic polypropylene (iPP) (PP 505P, SABIC, Riyadh, Saudi Arabia) was used. The melt flow rate (MFR) was determined in a previous study to be 2.0 g/10 min, with a density of 0.905 g/cm³ at 239 °C and 2.16 kg at room temperature [10]. The material was used as received and no purification was conducted.

### 2.2. Differential Scanning Calorimeter (DSC)

The differential scanning calorimeter (DSC Q2000, TA Instruments, New Castle, DE, USA) was used to measure the non-isothermal crystallisation process at five cooling rates of 2, 5, 10, 12.4 and 20 °C/min. The sample was heated at five rates of 2, 5, 10, 12.4 and 20 °C/min to 260 °C followed by a 180 s isothermal period at 260 °C, then cooled to 40 °C and held for another 180 s at 40 °C. During the measurement, a constant inert gas flow rate of 50 mL/min was set.

### 2.3. Flash DSC Measurements

For cooling rates much higher than the DSC Q2000 can apply, the Flash DSC 2+ by Mettler-Toledo, Columbus, OH, USA was used with five cooling rates of 600, 3000, 6000, 60,000 and 300,000 °C/min. The measurements determining the non-isothermal crystallisation process were set up using a heating rate equivalent to the following cooling rate. In addition, a 0.1 s isothermal period at 40 °C and at the starting temperature (230, 240, 250, 260 °C) was added between the heating and cooling processes to ensure thermal homogeneity of the sample. A small isothermal period was used here due to the low sample weight of ~25 ng, which was assumed to quickly reach a state of equilibrium. To determine the effect of the thermal history, the sample was heated up to four starting temperatures, followed by a cooling phase using a constant cooling rate of 6000 °C/min. All experiments used N_2_ as inert gas for cooling.

All measurements described above were non-isothermal (dynamic) crystallisation processes with a constant cooling rate until 40 °C was reached. In order to measure isothermal crystallisation processes, the sample rapidly cooled (60,000 °C/min) to a temperature between 70 and 120 °C. The cooling was followed by an isothermal period of 300 s, where the crystallisation proceeded. Afterward, the sample was cooled to 40 °C and heated again to 260 °C, to perform the next isothermal crystallisation.

## 3. Results

**Figure 1:**
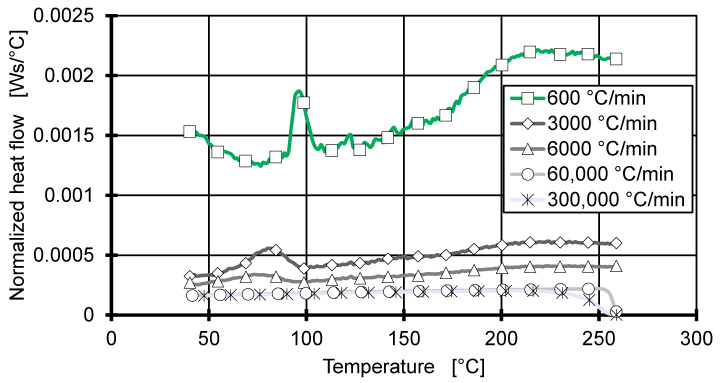
Flash DSC 2+ measurements of isotactic polypropylene at cooling rates from 600 to 300,000 °C/min normalized to their cooling rate.

Figure 1 shows the normalized heat flow measured by Flash DSC during cooling at five cooling rates. The normalized heat flow is the heat flow divided by the cooling rate; this allows a better identification of the exothermal crystallisation peaks. A significant crystallisation peak is observed for cooling rates of 6000 °C/min and lower. To reach the extremely high cooling rates (60,000 and 300,000 °C/min), the cooling rates are built up in the first few degrees, shown by a ramping normalized heat flow. However, the ramping of the cooling rate is negligible, since it stabilizes before the sample enters an undercooled state (<194 °C). The high cooling rates do not show exothermal crystallisation peaks. Here, the polymer is cooled too quickly, no crystallisation nucleus can form during the cooling period and the polymer solidifies amorphous. Since this work focuses on the crystallisation process on semi-crystalline thermoplastics, the measurements of 60,000 and 300,000 °C/min are neglected in the crystallisation kinetics analysis.

### 3.1. Avrami Exponent at High Cooling Rates

To describe the crystallisation of semi-crystalline thermoplastics, the Avrami model is widely used [18,26,27,28]. The model proposes a theoretical treatment of isothermal polymer crystallisation kinetics [17] and expresses the change in relative crystallinity ξ with time t as follows:

(1) $$\xi =1-\exp\left(-{\left(K_{a}t\right)}^{n_{a}}\right)$$

where $n_{a}$ is the Avrami exponent, which can take values in the interval 1 to 4 and characterises the spherulitic growth mechanism. $K_{a}$ is the isothermal crystallisation rate constant, containing nucleation and growth rates [18,25,29,30]. Equation (1) is the Avrami model in its most general form, which is used to characterise the crystallisation process by executing a parameter fit for $K_{a}$ and $n_{a}$ [30,31,32]. While homogeneous nucleation processes can only be thermal in nature and show an Avrami exponent of 4, heterogeneous nucleation processes may be either thermal or athermal showing an Avrami exponent of 3 [33]. However, both nucleation modes with an Avrami exponent equal or larger than 3 constitute a three-dimensional spherulitic growth, which is assumed for the measurements presented in this work [18,30]. The assumption is supported since the heterogeneous thermal nucleation is the most observed phenomenon in polymer crystallization, and Spekowius showed that the use of homogeneous nucleation leads to reasonable results within a microstructure simulation [19,30,34].

To account for non-isothermal crystallisation processes, the crystallisation rate constant should be adequately adjusted [35]. Since the DSC measurements are controlled at a constant cooling rate $\dot{T}$, the non-isothermal crystallisation rate constant $K_{c}$ is given as follows [25]:

(2) $$\ln\left(K_{c}\right)=\ln\left(K_{a}^{n_{a}}\right)/\dot{T}$$

**Figure 2:**
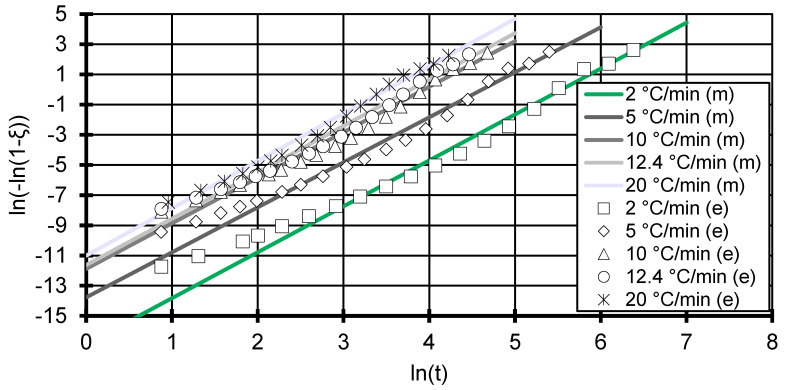
Avrami plot of 5 cooling rates from 2 to 20 °C/min. The markers show the experimental (e) data taken by the differential scanning calorimeter DSC Q2000, TA Instruments, New Castle, Delaware, USA. Lines represent the modelled (m) Avrami relation for each cooling rate. The experimental data were reduced to 20 points to ensure clarity of the figure.

**Figure 3:**
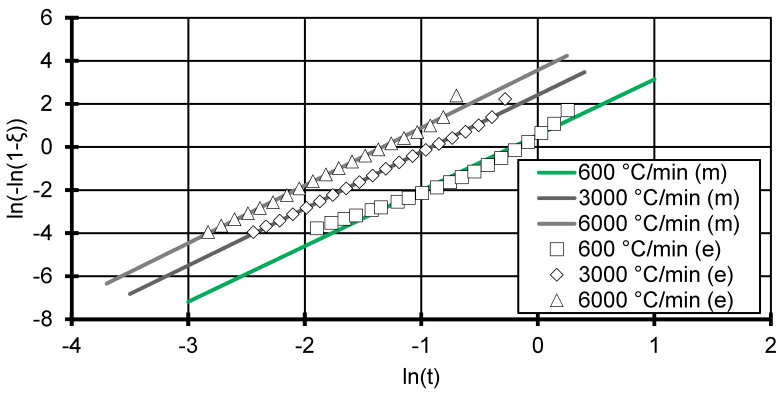
Avrami plot of 3 cooling rates from 600 to 6000 °C/min. The markers show the experimental (e) data taken by the Flash DSC 2+ by Mettler-Toledo, Columbus, Ohio, USA. Lines represent the modelled (m) Avrami relation for each cooling rate. The experimental data were reduced to 20 points to ensure clarity of the figure.

Subsequently, the corrected crystallisation rate constant and the Avrami exponent can be determined from a linear fit of an Avrami plot showing ln(−ln(1−ξ)) versus ln(t) (see Figure 2 and Figure 3) [17,25].

Due to the relative high measuring frequency of the DSC at smaller cooling rates and low signal-to-noise ratio, the Avrami plot can be drawn to very small y-values for the DSC measurements (see Figure 2). The Avrami model shows good fitting accuracy with R^2^ above 0.973 for all cooling rates in Figure 2 (see Table 1). However, the data deviates from the modelled slope systematically in that the data in the beginning of the crystallisation (ln(t)=1) start off slow. At this stage, nuclei are just forming and the crystallisation has not yet started off fully. Around ln(−ln(1−ξ))=−3 (equivalent to ~5% relative crystallinity), the experimental data curve upwards, which indicates a faster increase in relative crystallinity. This can also be observed in the Flash DSC measurements at the 600 °C/min curve but later in the curve (see Figure 3).

Since the Flash DSC measurements show a higher signal-to-noise ratio, values lower than ln(−ln(1−ξ))=−4 are not considered. This translates into the neglecting of measurements at the first 1.8% of solidification, which should not affect the fit given the amount of data points measured. The Flash DSC measurements for 3000 and 6000 °C/min show a more linear behaviour, but predict a smaller Avrami exponent and therefore point to different crystallisation processes.

The Avrami exponent at low cooling rates shows a stable value of 3 (±0.053), which is assumed and used by Mubarak et al. [18]. The measurements at cooling rates at and above 600 °C/min tend more towards an Avrami exponent between 2 and 3, which can be interpreted as disc-like (two-dimensional) crystal growth [24]. Due to the small sample size used in the Flash DSC combined with extremely high cooling rates, a crystal growth along the contact area between the sample and the Flash DSC sensor could be favoured. A truly three-dimensional growth is therefore slightly suppressed and leads to smaller Avrami exponents. Nevertheless, a three-dimensional growth is assumed across all cooling rates, which leads to the expectation of heterogeneous nucleation with spherical growth [24].

### 3.2. Hammami Model

Once the crystallisation process is characterised by the Avrami exponent, Mubarak et al. showed that the best model to predict the crystallisation process at different and especially high cooling rates is the model proposed by Hammami et al. [24], in combination with a correction for the non-isothermal induction time [18]. 

The Hammami model expresses the Avrami equation as follows:

(3) $$\xi =1-\exp\left(-\Psi \left(T\right)t^{n_{a}}\right)$$

where $\Psi \left(T\right)=4\pi /3*N*G{\left(T\right)}^{3}$ for the case of spherical growth and heterogeneous nucleation (Avrami exponent of 3) [36]. The crystallisation rate constant was identified to be dependent on the temperature T and proportional to the nucleation density N and the spherulitic growth function G(T) proposed by Hoffman and Lauritzen [37] via the following:

(4) $$G\left(T\right)=G_{0}\exp\left(\frac{-U^{*}}{R\left(T-T_{\infty }\right)}\right)\exp\left(\frac{-K_{g}}{fTT_{d}}\right)$$

where $G_{0}$ and $K_{g}$ are material parameters; $U^{*}$ is the activation energy for the segmental jump rate in polymers; $T_{\infty }=T_{g}-50\;K$ with $T_{g}$ being the glass transition temperature; the undercooling $T_{d}=T_{m,0}-T$, where $T_{m,0}$ is the equilibrium melting temperature; and f is a correction factor to implement a reduction in latent heat of fusion for undercooled melts [36,38]:

(5) $$f=\frac{2T}{\left(T_{m,0}+T\right)}$$

Assuming the temperature dependency of Ψ(T) is overwhelmingly influenced by the growth rate, the expression from Hammami et al. is adopted [24]:

(6) $$\Psi \left(T\right)≅C_{1}\exp\left(\frac{-3U^{*}}{R\left(T-T_{\infty }\right)}\right)\exp\left(\frac{-3C_{2}}{fTT_{d}}\right)$$

The time t used in the Avrami model and the Hammami model correlates with the cooling rate due to the non-isothermal treatment of the crystallisation. Therefore, the reference time is chosen to be at the crossing point of the equilibrium melting temperature:

(7) $$t=\frac{T_{m,0}-T}{\dot{T}}$$

where $t\left(T_{m,0}\right)=0$, which is in contrast to the interpretation in Mubarak et al., where $T_{m,0}$ is interpreted as the starting temperature from which the sample is cooled [18]. 

**Figure 4:**
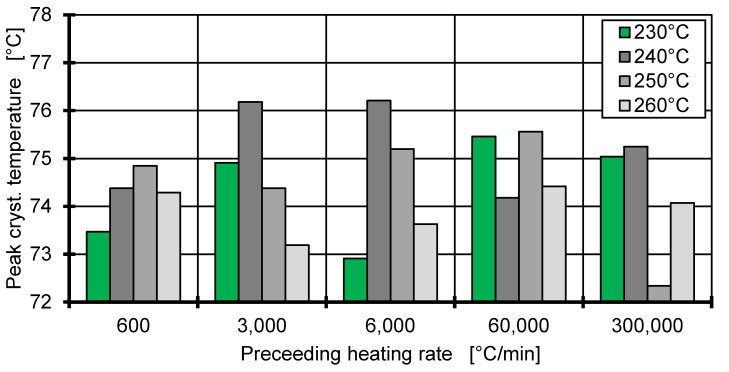
Peak crystallisation temperature at a cooling rate of 6000 °C/min with different starting temperatures and heating rates.

Figure 4 shows the influence of thermal history on the crystallisation temperature. The sample is cooled using a fixed cooling rate of 6000 °C/min but with preceding heating rates ranging from 600 to 300,000 °C/min and varying starting temperatures (230–260 °C). The interpretation of $T_{m,0}$ as starting temperature would show a linear dependency of the crystallisation temperature given the time until crystallisation does not change. However, Figure 4 shows a noisy but constant crystallisation temperature, which is independent of the starting temperature. After each heating process, a 0.1 s isothermal period at the starting temperature is implemented, to ensure thermal homogeneity of the sample. This seems to negate any effect that changing the heating rate might have. Therefore, the model is simplified by adopting the equilibrium melting temperature (194 °C for iPP [30]) as the reference temperature and neglecting the preceding heating rate.

### 3.3. Non-Isothermal Induction Time

The Avrami model uses the beginning of the crystallisation ($T=T_{m,0}$) as the reference time. However, between the time of reaching $T_{m,0}$ in a non-isothermal cooling process and the first nucleus emerging within the sample, a certain amount of time elapses, which is called induction time [18,29]. The temperature at the induction time is the transition/crystallisation temperature $T\left(t_{I}\right)=T_{c}$, which is the onset temperature of the exothermal crystallisation peak. 

Due to the non-isothermal treatment, the non-isothermal induction time $t_{I}$ is itself dependent on the cooling rate:

(8) $$t_{I}=\frac{T_{m,0}-T_{c}}{\dot{T}}$$

It is necessary to include this effect in a non-isothermal kinetics model, since Patel et al. [23] found that an over-prediction of the crystallisation temperature in non-isothermal models can be attributed to the fact that the induction time is neglected. The non-isothermal induction time is derived from the isothermal induction time $t_{i}$ by integrating over all isothermal induction times up to the non-isothermal induction time by applying the Sifleet method [39]:

(9) $$1=\int_{0}^{t_{I}}\frac{dt}{t_{i}\left(T\right)}$$

**Figure 5:**
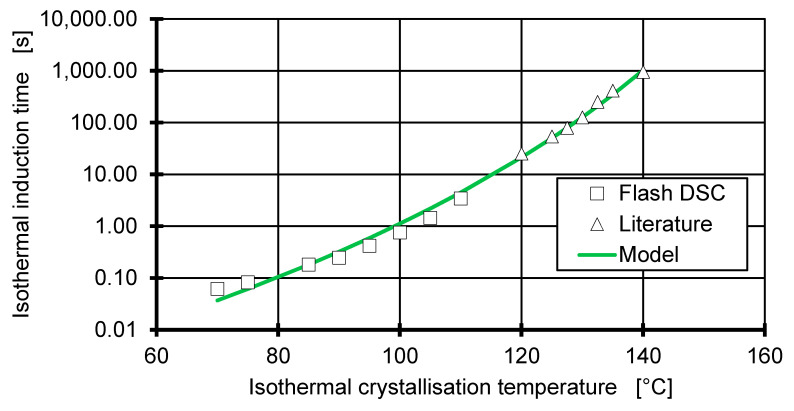
Combining measurements of the isothermal induction time from the literature (∆) [18] and Flash DSC measurements (□) to fit both parameters of the Godovsky isothermal induction time model.

Godovsky et al. [40] proposed the isothermal induction time to be modelled as follows:

(10) $$t_{i}=t_{m}{\left(T_{m,0}-T\right)}^{-\alpha }$$

where $t_{m}$ and α are material parameters. The measurement of the isothermal induction time is performed via Flash DSC at a cooling rate of 60,000 °C/min, rapidly cooling the sample to temperatures between 70 and 120 °C and then held for 10 s. The emerging isothermal crystallisation peak is then used to determine the isothermal induction time. Using Equation (10), the parameters are fitted to $t_{m}=2.177\times 10^{24}\;s/{}^{\circ}C^{a}$ and α=12.31 with the equilibrium melting temperature $T_{m,0}=194\;C$. Figure 5 shows the prediction of the isothermal induction time against the data created using the Flash DSC and measurements done by Mubarak et al. [18]. The model shows good agreement with the measured data (R^2^ = 0.993) and data found in the literature [18].

The calculation of the non-isothermal induction time in Equation (9) can be simplified using the approach of Isayev et al. [29] to the following:

(11) $$1=\overset{t_{I}}{\underset{0}{\sum }}\frac{1}{t_{m}{\left(T_{m,0}-T\right)}^{-\alpha }}\Delta t$$

and applying Equation (8) yields the following:

(12) $$1=\overset{T_{c}}{\underset{T_{m,0}}{\sum }}\frac{1}{t_{m}{\left(T_{m,0}-T\right)}^{-\alpha }}\frac{\Delta T}{\dot{T}}$$

where $T_{c}$ is the crystallisation temperature and a constant cooling rate $\dot{T}$ is assumed. Due to the nature of the DSC and Flash DSC measurements, the assumption of a constant cooling rate applies to all measured crystallisation data. Equation (12) can be simplified by defining:

(13) $$T=T_{j}=T_{m,0}-\Delta T*j$$

(14) $$\Delta T=\frac{T_{m,0}-T_{c}}{n};n\in \mathbb{N}$$

which yields:

(15) $$1=\frac{\Delta T^{\alpha +1}}{t_{m}\dot{T}}\overset{n}{\underset{j=0}{\sum }}j^{\alpha }$$

where j is the temperature increment counter from 0 to n, and $T_{c}=T_{m,0}-\Delta T*n$. ΔT corresponds to the accuracy of the model and is chosen to be ΔT = 0.01 K. The modified Hammami equation then results in the following:

(16) $$\xi =1-\exp\left(-\Psi \left(T\right){\left(\frac{T_{c}-T}{\dot{T}}\right)}^{n_{a}}\right)$$

Equation (16) is only valid for $T\leq T_{c}$ due to numerical considerations of ξ, where negative values have no physical meaning.

### 3.4. Application of the Hammami Model

**Figure 6:**
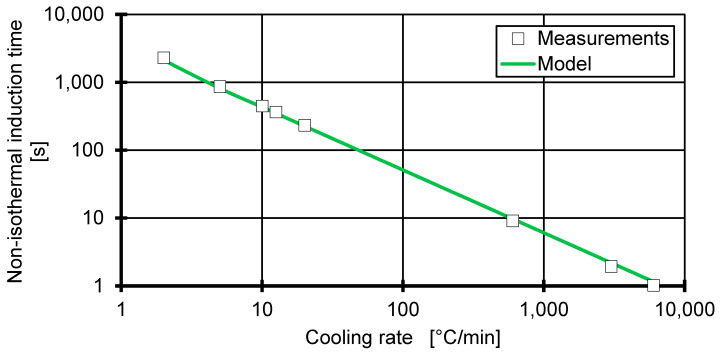
The markers show the time to reach the onset crystallisation temperature in a non-isothermal cooling with constant cooling rate. The time reference (t=0) is chosen to be the crossing point of the equilibrium melting temperature.

The non-isothermal induction time is calculated from Equation (11) via Equation (15) by using a and $t_{m}$ calculated from the isothermal induction time. Figure 6 shows the modelled non-isothermal induction time. This is in good comparison with the measured non-isothermal induction time, which is the time measured from $T_{m,0}$ to the onset temperature of the crystallisation peak. Therefore, the Isayev approach can be used to predict non-isothermal induction times up to 6000 °C/min. However, the formulation can only be used as long as the cooling rate allows crystallisation to occur.

Since the Avrami exponent (see Table 1) and the non-isothermal induction time are both in good agreement with the experimental data, the Hammami model can be applied to predict the relative crystallinity over time for each of the cooling rates between 2 and 6000 °C/min. This is done by performing a parameter fit for $C_{1}$ and $C_{2}$ with $U^{*}$ = 17,200 J/mol and $T_{\infty }$ = −61.6 °C for each cooling rate (see Table 2) [30].

For each cooling rate, an individual set of parameters is fitted, which shows the influence of the cooling rate on the fit parameters. $C_{1}$ is linked to the growth rate constant from the Hoffman–Lauritzen theory (HL), which is usually assumed to be a material-specific constant. However, a clear dependence on the cooling rate is observed. Since the natural logarithm of $C_{1}$ is shown in Table 2, it needs to be pointed out that the difference in growth rate constant are many orders of magnitude. This leads to the conclusion that the Hammami model is not suitable to predict the crystallisation at extremely high cooling rates. However, the results at low cooling rates (up to 20 °C/min) are comparable to the proven results in Mubarak et al. [18]. 

**Figure 7:**
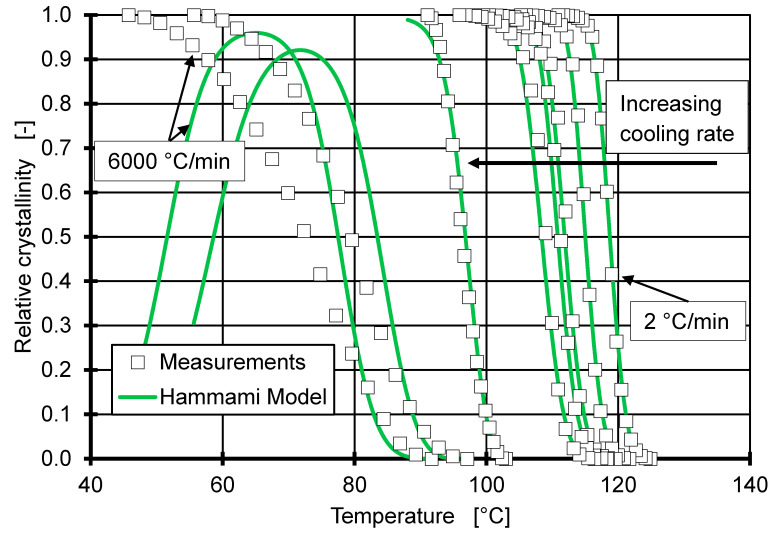
Hammami model using the individual fitted parameters for each cooling as mentioned in Table 2. The graph most to the left shows the crystallisation process for a cooling rate of 6000 °C/min, whereas the graph most to the right shows the crystallisation process for a cooling rate of 2 °C/min.

Figure 7 shows the data from the individual fitted Hammami model in comparison to the experimental data for each cooling rate. The comparable low R^2^ value for high cooling rates relates to a failure to predict the crystallisation process. For the cooling rates of 3000 and 6000 °C/min, the model predicts a rise in relative crystallinity up to ~90% followed by remelting of the crystals for continued cooling, which is not realistic. However, up to the cooling rate of 600 °C/min, the Hammami model seems to be applicable and in good agreement with the experimental data and can be compared to the results of the CTD model.

The reason for the deviation at cooling rates above 600 °C/min can be attributed to the correction factor f (Equation (5)), which was introduced to the isothermal HL to have validity over a large range of undercooling. The validity of the application of the factor in a non-isothermal approach is questionable, since it originates from an isothermal treatment and was tested at an undercooling much smaller than 100 °C. Furthermore, the thermodynamic treatment of the crystallisation process within HL is based on quiescent conditions present during cooling. It can be stated from the results in Figure 7 that the cooling of iPP with 3000 °C/min and above can no longer be treated as quiescent conditions. In order to enable a precise description of crystallization at all injection moulding-relevant cooling rates, a model for dynamic processes must be developed from thermodynamics at non-quiescent conditions. 

Furthermore, the HL expects crystallisation to occur at some point during the cooling phase. However, if semi-crystalline thermoplastics are cooled sufficiently fast (60,000 °C/min for iPP), they solidify purely amorphous. All cooling rates used in Figure 7 show a significant exothermal peak during cooling, therefore the HL applies. However, the HL neglects the absolute amount of crystallized material in relation to amorphous material (crystallization degree) during the solidification. To correctly predict the crystallisation process at high cooling rates, it is necessary to transform the crystallisation model to a solidification model, which correctly predicts the crystallisation degree. This would even allow the modelling of purely amorphous solidification at cooling rates experienced in injection moulding processes and will be part of further research.

## 4. Continuous Two-Domain pvT Model

The continuous two-domain pvT model (CTD) by Wang et al. [10] is a derivation of the empirical Tait model. It removes the discontinuity of the original formulation around the transition temperature by forcing the models, which describe the solid and molten states, to intersect at $T_{c}$. This stabilizes the specific volume prediction during the injection moulding simulation [9]. In addition, the CTD introduces a cooling rate-dependent term q influencing the specific volume dependent on pressure $\nu_{c}\left(p,q\right)$ and the transition temperature as a function of pressure $T_{c}\left(p,q\right)$ [10]. Chang et al. [39] proposed a logarithmic dependency of $T_{c}$ on the cooling rate, which is implemented in the following form:

(17) $$\ln\left(q\right)=\ln\left(\frac{\dot{T}}{\dot{T_{0}}}+1\right)$$

where $\dot{T_{0}}$ is a reference cooling rate set to 2 °C/min to eliminate the units within the logarithmic expression. The relation used within the CTD for the transition temperature results in the following:

(18) $$T_{c}\left(p,q\right)=T_{0}-b_{1}*\ln\left(q\right)+b_{2}*p+b_{3}*p^{2}$$

**Figure 8:**
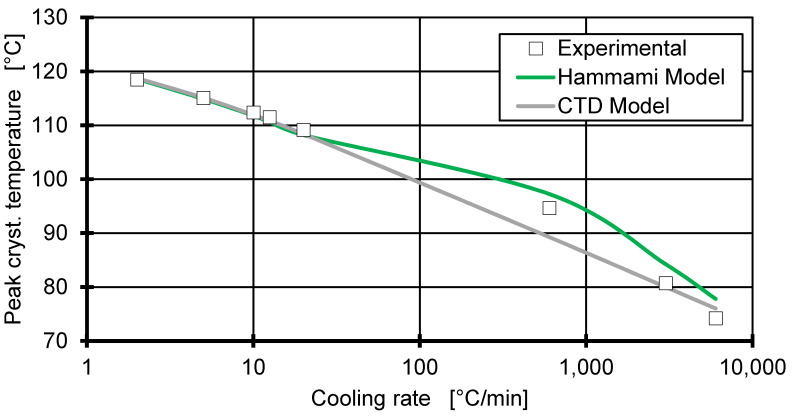
Comparison between the prediction of the peak crystallisation temperature by the Hammami model and the continuous two-domain pvT model (CDT). The markers show the measured peak crystallisation temperatures from the DSC and Flash DSC experimental data.

Since the peak crystallisation temperature corresponds to the transition point of the pvT measurement, the peak crystallisation temperatures are calculated from the prediction of the relative crystallinity. By assuming the peak crystallisation temperature at the peak of the change in relative crystallinity, the Hammami model is used to predict $T_{c,peak}$ by evaluating Δξ/ΔT for its maximum (see Figure 8). In parallel, a parameter fit for $T_{c}\left(p,q\right)$ (Equation (18)) is performed using the combined data of Flash DSC and DSC measurements, as well as pvT measurements (performed in [10]). The fit yields the following:

(19) $$T_{c}\left(p,q\right)=122.73-5.76*\ln\left(q\right)+0.023*p+1.48*10^{-6}*p^{2}$$

As seen in Figure 8, the CTD fit is in high agreement with the experimental data, thereby validating the assumption of a logarithmic relationship between $T_{c}$ and q for high cooling rates. Due to the individual fitting of each crystallisation process for the Hammami model, it should show the best agreement with the experimental data. However, since the model cannot make accurate predictions of the crystallisation process at high cooling rates, the crystallisation temperature is not accurately predicted. Both models show high agreement for low cooling rates; this was to be expected, since they were developed for this regime, but the CTD model is able to predict crystallisation temperature up to 6000 °C/min, neglecting the outlier at 600 °C/min. This should allow the CTD model to predict the pvT behaviour at high cooling rates and high pressures. This is however challenging to validate experimentally.

The good prediction of the transition temperature by the CTD would allow the integration of the pressure dependency into the fit parameters of the Hammami model. Due to the issues of the Hammami model formulation that we have pointed out, a combination is not feasible until these issues are resolved for high cooling rates.

## 5. Conclusions and Outlook

Since temperature conditions in injection moulding processes are mainly non-isothermal and cooling rates reach from 3000 °C/min at the mould walls to 60 °C/min near the core of the part, it is necessary to have a model predicting the crystallisation behaviour at a wide range of cooling rates [11].

The model presented by Hammami et al. [24], which is based on the Hoffman–Lauritzen theory and the Avrami model, was applied to predict the crystallisation process of isotactic polypropylene and showed high prediction accuracy up to 600 °C/min. In order to use the Hammami model, the Avrami exponent and the non-isothermal induction time were determined in preliminary evaluations. This showed that the Isayev approach to determine the non-isothermal induction time could be applied to all cooling rates showing crystallisation (up to 6000 °C/min). The Avrami plot showed even better linearity for the cooling rates >600 °C/min, but at a reduced value. Despite the accuracy shown by the Avrami plot and the prediction of the non-isothermal induction time, the Hammami model formulation is not suitable for cooling rates above 600 °C/min.

Solidification in injection moulding processes usually occurs at pressures above 200 bar. This work tried to combine the continuous two-domain pvT model (CTD) by Wang et al. [10] with the prediction of transition temperature of the Hammami model. The agreement of both models at the pressure of 1 bar allows a combination of both models in a pressure-dependent crystallisation kinetics model. In a first step, the prediction of the crystallisation temperature by the Hammami model up to a cooling rate of 600 °C/min was used to compare it to the CTD model at 1 bar pressure.

It was shown that the logarithmic cooling rate dependency of the transition temperature assumed in CTD was valid for all used cooling rates and showed a higher fitting accuracy than the Hammami model. However, using the crystallisation data, the CTD was expanded for low pressures and high cooling rates. Fitting all available data on the transition temperature of the CTD yields an accurate prediction of the pressure and cooling rate dependency of the transition temperature with R^2^ = 0.993. However, due to the low fitting accuracy of the Hammami model at process-relevant cooling rates, a pressure dependency of the material parameters has not yet been derived but will be part of future research.

## Figures and Tables

**Table 1:** Results of the linear fit of the Avrami plots of each individual cooling rate. All fits show good fitting accuracy with an R^2^ value around 0.97.

Avrami Parameters	Cooling Rate (°C/min)							
	2	5	10	12.4	20	600	3000	6000
$n_{a}$	3.044	2.983	3.017	3.073	3.141	2.580	2.640	2.678
$\ln\left(K_{c}\right)$	−16.87	−13.77	−11.88	−11.63	−11.01	0.55	2.42	3.57
$\ln\left(K_{a}^{n_{a}}\right)$	−0.51	−1.14	−1.97	−2.41	−3.67	5.54	120.8	355.6
R^2^	0.9771	0.9734	0.9773	0.9770	0.9843	0.9693	0.9977	0.9955

**Table 2:** Fit parameters for the Hammami model to predict the relative crystallinity at different cooling rates. The R^2^ values for the fits are high for the low cooling rates. However, for high cooling rates the model loses accuracy.

Fit Parameters	Cooling Rate (°C/min)							
	2	5	10	12.4	20	600	3000	6000
$\ln\left(C_{1}\right)$	49.9	59.2	60.7	64.8	64.5	73.4	112.1	144.7
$C_{2}*10^{-5}$	2.81	3.48	3.48	3.84	3.72	3.52	7.46	11.08
R^2^	0.97	0.97	0.96	0.97	0.96	0.91	0.92	0.94

## Abbreviations

CTD	Continuous two-domain pvT model by Wang et al.
DSC	Differential scanning calorimetry
HL	Hoffman–Lauritzen theory
iPP	Isotactic polypropylene
MFR	Melt flow rate
pvT	Correlations between pressure, specific volume and temperature of polymers

## References

[1] Sun X., Su X., Tibbenham P., Mao J., Tao J. (2016). The application of modified PVT data on the warpage prediction of injection molded part. J. Polym. Res..

[2] Huang C., Hsu Y., Chen B. (2019). Investigation on the internal mechanism of the deviation between numerical simulation and experiments in injection molding product development. Polym. Test..

[3] Heidari B.S., Davachi S.M., Moghaddam A.H., Seyfi J., Hejazi I., Sahraeian R., Rashedi H. (2018). Optimization simulated injection molding process for ultrahigh molecular weight polyethylene nanocomposite hip liner using response surface methodology and simulation of mechanical behavior. J. Mech. Behav. Biomed. Mater..

[4] Wang J. (2012). PVT properties of polymers for injection molding. Some Critical Issues for Injection Molding.

[5] Rodgers P.A. (1993). Pressure–volume–temperature relationships for polymeric liquids: A review of equations of state and their characteristic parameters for 56 polymers. J. Appl. Polym. Sci..

[6] Júnior P., José E., Soares R.D.P., Cardozo N.S.M. (2015). Analysis of equations of state for polymers. Polímeros.

[7] Yi Y.X., Zoller P. (1993). An experimental and theoretical study of the PVT equation of state of butadiene and isoprene elastomers to 200 °C and 200 MPa. J. Polym. Sci. Part B Polym. Phys..

[8] Song M., Qin Q., Zhu J., Yu G., Wu S., Jiao M. (2019). Pressure-volume-temperature properties and thermophysical analyses of AO-60/NBR composites. Polym. Eng. Sci..

[9] Wang J., Hopmann C., Schmitz M., Hohlweck T., Wipperfürth J. (2019). Modeling of pvT behavior of semi-crystalline polymer based on the two-domain Tait equation of state for injection molding. Mater. Des..

[10] Wang J., Hopmann C., Röbig M., Hohlweck T., Kahve C., Alms J. (2020). Continuous Two-Domain Equations of State for the Description of the Pressure-Specific Volume-Temperature Behaviour of Polymers. Polymer.

[11] Suárez S.A., Naranjo A., López I.D., Ortiz J.C. (2015). Analytical review of some relevant methods and devices for the determination of the specific volume on thermoplastic polymers under processing conditions. Polym. Test..

[12] Zuidema H., Peters G.W.M., Meijer H.E.H. (2001). Influence of cooling rate on pVT-data of semicrystalline polymers. J. Appl. Polym. Sci..

[13] Van Drongelen M., Van Erp T.B., Peters G.W.M. (2012). Quantification of non-isothermal, multi-phase crystallization of isotactic polypropylene: The influence of cooling rate and pressure. Polymer.

[14] Xie P., Yang H., Cai T., Li Z., Li Y., Yang W. (2018). Study on the pressure-volume-temperature properties of polypropylene at various cooling and shear rates. Polym. Korea.

[15] Wang J., Hopmann C., Schmitz M., Hohlweck T. (2020). Process dependence of pressure-specific volume-temperature measurement for amorphous polymer: Acrylonitrile-butadiene-styrene. Polym. Test..

[16] Wang J., Hopmann C., Schmitz M., Hohlweck T. (2019). Influence of measurement processes on pressure-specific volume-temperature relationships of semi-crystalline polymer: Polypropylene. Polym. Test..

[17] Avrami M. (1940). Kinetics of phase change. II transformation-time relations for random distribution of nuclei. J. Chem. Phys..

[18] Mubarak Y., Harkin-Jones E.M.A., Martin P.J., Ahmad M. (2001). Modeling of non-isothermal crystallization kinetics of isotactic polypropylene. Polymer.

[19] Nakamura K., Katayama K., Amano T. (1973). Some aspects of nonisothermal crystallization of polymers. II. Consideration of the isokinetic condition. J. Appl. Polym. Sci..

[20] Ozawa T. (1971). Kinetics of non-isothermal crystallization. Polymer.

[21] Ding Z., Spruiell J.E. (1997). Interpretation of the nonisothermal crystallization kinetics of polypropylene using a power law nucleation rate function. J. Polym. Sci. Part B Polym. Phys..

[22] Kamal M.R., Chu E. (1983). Isothermal and nonisothermal crystallization of polyethylene. Polym. Eng. Sci..

[23] Patel R.M., Spruiell J.E. (1991). Crystallization kinetics during polymer processing–analysis of available approaches for process modeling. Polym. Eng. Sci..

[24] Hammami A., Spruiell J.E., Mehrotra A.K. (1995). Quiescent nonisothermal crystallization kinetics of isotactic polypropylenes. Polym. Eng. Sci..

[25] Hao W., Yang W., Cai H., Huang Y. (2010). Non-isothermal crystallization kinetics of polypropylene/silicon nitride nanocomposites. Polym. Test..

[26] Liu X., Wu Q. (2002). Non-isothermal crystallization behaviors of polyamide 6/clay nanocomposites. Eur. Polym. J..

[27] Somrang N., Nithitanakul M., Grady B.P., Supaphol P. (2004). Non-isothermal melt crystallization kinetics for ethylene–acrylic acid copolymers and ethylene–methyl acrylate–acrylic acid terpolymers. Eur. Polym. J..

[28] Jiasheng Q., Pingsheng H. (2003). Non-isothermal crystallization of HDPE/nano-SiO 2 composite. J. Mater. Sci..

[29] Isayev A.I., Chan T.W., Shimojo K., Gmerek M. (1995). Injection molding of semicrystalline polymers. I. Material characterization. J. Appl. Polym. Sci..

[30] Spekowius M. (2017). A New Microscale Model for the Description of Crystallization of Semi-crystalline. Ph.D. Thesis.

[31] Celli A., Zanotto E.D. (1995). Polymer crystallization: Fold surface free energy determination by different thermal analysis techniques. Thermochim. Acta.

[32] Yuryev Y., Wood-Adams P. (2010). A Monte Carlo Simulation of Homogeneous Crystallization in Confined Spaces: Effect of Crystallization Kinetics on the Avrami Exponent. Macromol. Theory Simul..

[33] Wunderlich B. (1976). Crystal nucleation, growth, annealing. Macromolecular Physics, Volume 2.

[34] Falkai V.B.V. (1960). Schmelz-und kristallisationserscheinungen bei makromolekularen substanzen. I. Kristallisationskinetische untersuchungen an isotaktischem polypropylen. Makromol. Chem. Macromol. Chem. Phys..

[35] Jeziorny A. (1978). Parameters characterizing the kinetics of the non-isothermal crystallization of poly (ethylene terephthalate) determined by DSC. Polymer.

[36] Elias H.G. (1977). Chemische Struktur und Synthese. Makromoleküle, Band 1.

[37] Hoffman J.D., Davis G.T., Lauritzen J.I. (1976). The rate of crystallization of linear polymers with chain folding. Treatise on Solid State Chemistry.

[38] Lauritzen J.I., Hoffman J.D. (1960). Theory of formation of polymer crystals with folded chains in dilute solution. J. Res. Natl. Bur. Stand. Sect. A Phys. Chem..

[39] Sifleet W.L., Dinos N., Collier J.R. (1973). Unsteady-state heat transfer in a crystallizing polymer. Polym. Eng. Sci..

[40] Godovsky Y.K., Slonimsky G.L. (1974). Kinetics of polymer crystallization from the melt (calorimetric approach). J. Polym. Sci. Polym. Phys. Ed..

